# Digital media and misinformation: An outlook on multidisciplinary strategies against manipulation

**DOI:** 10.1007/s42001-021-00118-8

**Published:** 2021-05-27

**Authors:** Danielle Caled, Mário J. Silva

**Affiliations:** INESC-ID, Instituto Superior Técnico, Universidade de Lisboa, R. Alves Redol 9, 1000-029 Lisbon, Portugal

**Keywords:** Digital misinformation, Disinformation, Fake news, Digital media, Fact-checking

## Abstract

This review discusses the dynamic mechanisms of misinformation creation and spreading used in social networks. It includes: (1) a conceptualization of misinformation and related terms, such as rumors and disinformation; (2) an analysis of the cognitive vulnerabilities that hinder the correction of the effects of an inaccurate narrative already assimilated; and (3) an interdisciplinary discussion on different strategies for coping with misinformation. The discussion encompasses journalistic, educational, governmental and computational viewpoints on the topic. The review also surveys how digital platforms handle misinformation and gives an outlook on opportunities to address it in light of the presented viewpoints.

## Introduction

In the last years, social media and alternative news sources, which allow any user to produce unverified online content, have gained popularity. Social networks can break down physical barriers by connecting geographically dispersed people, easing political and economic constraints. Their rise has changed the paradigm of information production and consumption, as they are now the preferred means of staying up to date with news and current affairs [1]. While before the reader was a passive agent waiting for the asynchronous production of news, there is now an increasing demand for timely, near real-time, journalistic coverage. As news organizations cannot meet this demand, the public itself takes their role. The intrinsic dynamism of social networks is able to fill this gap, giving therefore voice to the production of amateur content. Social media platforms offer an environment suited for collective sense-making and for the emergence of stories created with the intention of filling information gaps left by mainstream media [2, 3].

However, the new social media also enable real-time viralization of news stories. As consequence, they became a vehicle for the diffusion of misinformation to a global audience [4, 5]. As new content is continuously published and propagated, it is very hard not only for end users but even for professional journalists to check and validate all this material. Social media make verification even harder since it becomes difficult to track the original author and context of the post [6]. In addition to this new role of the public in the production of content, there is a growing demand for immediate coverage which pressures news organizations to publish unfinished or unverified material. It is not uncommon that a novel or viral event is first released on social networks and then absorbed by the mainstream media. The reliance on social networks as information sources combined to a predilection for sensationalism, the need for constant novelty, the lack of resources for fact-checking (i.e., investigating the veracity of a story) and the emphasis on profits over civic responsibility made the mainstream media susceptible to manipulation [7].

The above vulnerabilities are increasingly perceived by the readership of the traditional media [8, 9]. This represents a social issue since, with the loss of trust in the media, the public is less likely to have access to accurate information, compromising their critical judgment, their political knowledge, and the full exercise of democracy [7, 10, 11]. As citizens’ confidence in institutions declines and the credibility of official information decreases, the audience tends to spiral into alternative sources of information [9].

Fact-checking is generally conducted from the journalistic perspective, pipelining gathered facts through a quality news production process before eventually publishing the news for consumption by their readership. Social platforms address this issue from the same viewpoint, scaling up the number of partner journalist organizations across the world to validate the content piped through their information streams [12]. These outlets flag misinformation, which in turn is used to downrank “bad” postings. However, as new content is also continuously published and propagated through the Web, it is impossible for journalists to check and validate all this material in time even when armed with the best available tools. Moreover, when internet users access publishers directly instead of being mediated by a gatekeeper, they are defenseless.

In view of the characteristics and mechanisms for the dissemination of misinformation, we conclude that only through multidisciplinary responses, misinformation can be contained. However, although many previous studies address misinformation, most of them only consider a single aspect or a particular perspective to deal with this problem. For example, Fallis carried a philosophical survey on disinformation, discussing various analysis proposed by information scientists and philosophers [13]. Wardle and Derakhshan addressed the same topic from a communication perspective [14]. Other computational approaches explored the automatic detection of misinformation [15–17], lacking an interdisciplinary look on the topic.

This survey proposes a systematic review with emphasis on exploring interdisciplinary paradigms and the different strategies that have been used to contain misinformation spread. Through the analysis of the existing literature, five main approaches were identified, systematized, and characterized through examples of guidelines, actions, projects and systems designed to curb misinformation. The analysis comprises perspectives on:

- journalism;
- education;
- governmental responses;
- computational solutions; and
- digital platforms.

This paper contributes to enhance our understanding of misinformation mechanisms, formulating an integrated view and discussing interdisciplinary and complementary responses, thus facilitating future research on a more holistic theoretical framework on the topic.

The next section presents a conceptualization of misinformation and discusses the dynamics of misinformation spread. The subsequent section sheds light on the cognitive phenomena that make audiences more vulnerable to misinformation. Then, different strategies for coping with misinformation are presented and discussed. Finally, we conclude this survey with a critical summary and a future outlook on opportunities.

## Misinformation conceptualization

Many terms have been used to refer to content whose veracity is false or unknown. Buzzwords, like *misinformation*, *disinformation*, *hoax*, *rumor,* and *fake news* are employed interchangeably and shallowly. Since these concepts are sometimes conflicting, the nomenclature adopted in this review must be clarified. We begin by defining *news* as a set of asserted “claims, statements, speeches, posts, among other types of information” of public interest [18]. With this definition, the news concept goes beyond the journalistic production, also encompassing posts on social networks.

*Misinformation* is a macro-concept, indicating misleading or inaccurate information, that can be manifested in different shapes (see Table 1). Although misinformation is not necessarily associated with the intention to deceive, it is related to the dissemination of false or incorrect narratives, which can occur, for example, due to lack of information, misunderstanding of a message or even distortion of information for humorous purposes. Misinformation is multi-modal and can be disseminated through images, videos, audio, and text; or even by combining these modalities. Saez-Trumper lists two groups of mechanisms for the dissemination of misinformation: social and technological attacks [19]. Social attacks are mechanisms that exploit weaknesses in information systems through human resources, whether these are individual or collective agents (e.g., sock-puppeting and click farms). Technological attacks, on the other hand, depend on computational resources, such as the creation of social bots or deepfake techniques (See Table 2).

This review places a higher emphasis on *disinformation* and *rumors* since these concepts are closely related and may even be considered different nuances of the same problem. For a more detailed typology of the misinformation ecosystem, refer to Zannettou et al. [20].

**Table 1:** Different shapes of misinformation

Disinfor-mation	False information intended to mislead. Disinformation amplifiers do not always generate it intentionally, e.g., news organizations or social media are frequently manipulated by deceivers to disseminate inaccurate or misleading information [21].
Rumor	Defined as a piece of information whose “veracity status is yet to be verified at the time of posting” [15]. A rumor may not necessarily report a false story, it may indeed be later confirmed as true. What really characterizes a rumor is the insecure basis of its evidence [22].
Clickbait	Used to attract a greater flow of readers to websites through provocative and catchy headlines, appealing to users’ curiosity, and luring them to click on links that do not deliver what was promised [23]. The use of exaggerated titles that prompt to disappointing content is a common characteristic of the clickbaits. The main motivation for using clickbaits is the conversion of traffic into revenue (website monetization).
Satirical News	Use of sarcasm and irony to provoke laughter or mockery to entertain the reader; relies on unexpectedness, frequently entailing a combination of incompatible entities and/or ideas [24]. While it is assumed that consumers of satirical news are aware of the humorous intent of the stories, such narratives can spread misinformation and induce confusion in the audience [17].
Social Spam	Different kinds of attacks (e.g., phishing, spreading of advertising messages and viruses) promoted by malicious agents [16, 25]. Social spam approaches are characterized by two strategies: (1) spammers are able to adapt their spamming patterns to avoid being discovered; and (2) spammers who pretend to be normal users interact with their peers, creating a social network for establishing a chain of influence [26].

**Table 2:** Following the typology proposed by Saez-Trumper [19] and adding the definitions proposed by Fernquist et al. [27], a description of the most popular mechanisms to spread online misinformation

Type	Technique	Description
Social	Astroturfing	A practice of disguising the sponsors of a message to give the impression that it originated spontaneously, representing the public interest and community concerns.
	Circular reporting	Information, originated by a single source, appearing to come from multiple independent sources and channels with minor modifications.
	Click farms	An operation in which a large group fraudulently interacts with a website to artificially boost internet traffic, deceiving online systems.
	Data voids	Manipulations exploring the lack of natural content to induce search engines to return low-quality and problematic content [28].
	Sock-puppets	The use of false or misleading identities on the Internet to interact with ordinary users on social media for purposes of deception.
	Web brigades	A set of users coordinated to undertake large-scale disinformation campaigns by exploiting the weakness of communities and systems.
Technical	Deepfakes	Manipulations created using deep learning techniques trained on a large number of samples to automatically map facial expressions and to achieve face swapping [29, 30].
	Spam bots	Bots designed to post on online comment sections, spread advertisements, or for extracting contact information for spam mailing lists.
	Social bots	Bots designed to automatically spread messages and advocate ideas, thus influencing public opinion on a given topic. They can also create fake accounts and simulate the popularity of social media profiles (e.g., through a massive network of followers).
Hybrid	Cyborgs	Hybrid accounts combining automatic and human curation. In such accounts, a human periodically takes over a bot account in order to disguise and increase the account’s credibility.
	Sybils	Impersonators who try to connect with a real user’s friends and take advantage of its reputation [31]. Sybils accounts may be operated by bots to spread disinformation and reach a wider audience.

### Disinformation

Disinformation is usually associated with governmental or military activity, but organizations (e.g., news services) and single individuals also act like disinformation sources [21]. Disinformation actors operate in blogs and websites, forums and message boards, mainstream social media sites and online communities, targeting the intrinsic vulnerabilities of the news media ecosystem to increase the visibility of their messages and, thus, achieve a wider audience [7]. Disinformation is closely related to *fake news*, which is also used for denoting false news stories that are published as if they were genuine [32]. However, after gaining much evidence in recent years, *fake news* became an ambiguous and contested expression [7], distorted due political usage. Consequently, once any story conflicting with an ideology could be labelled as *fake*, the term *fake news* gained a new meaning, also being used to restrict and undermine the free press [14, 32, 33].

Previous works introduced two types of *disinformation*: *serious fabrications* and *large-scale hoaxes* [34]. *Serious fabrications* are fraudulent reports marked by a style trying to mimic the journalistic writing (e.g., alleged coverage of an event, fake interviews, pseudoscience articles) that often become viral [17]. *Large-scale hoaxes* are narratives built with the purpose of scamming the audience. Different from pranks or practical jokes, *hoaxes* are more complex structures which include deceptions that may cause harm or material loss [35]. A striking feature of hoaxes is that they are not limited to a single article, but spread out on large-scale, targeting public figures or ideologies [17].

### Rumors

Rumors arise from ambiguous, confusing, or unclear situations, when scarce information is available or as a product of untrusted information sources [22, 36]. Newsworthy events, especially during crisis situations, when the audience has little access to trusted evidence, are likely to produce unverified stories [37]. This fact is associated with the collective sense-making function of a rumor, as people discuss it to reach a group interpretation of their situational context [22]. Moreover, when the audience does not receive answers from official sources in a timely manner, they attempt to fill this information gaps with rumors [2]. Hence, the spread of rumors influences people’s perception and understanding of the event and can induce dangerous consequences for the society, namely dissemination of fear, hate or euphoria, causing protests, destruction of property, defamation of brands and public entities, and other undesired effects [38, 39]. Rumors are differentiated based on temporal characteristics: (1) *breaking news rumors* are unseen rumors that emerge in the context of breaking news stories and must be immediately debunked in order to avoid impulsive reactions from the audience; and (2) *long-standing rumors* are stories that arouse great interest and circulate for long periods until their veracity is revealed [15].

### Misinformation dynamics: from social media to news articles

Disinformation and rumor diffusion are both performed through similar strategies [7, 40]. In general, propagation is cyclic, beginning with media manipulators creating a juicy story of an alleged incident or event. The story can come in different formats: memes, false discourses, false images, false videos, and misleading content [41].

**Fig. 1:**
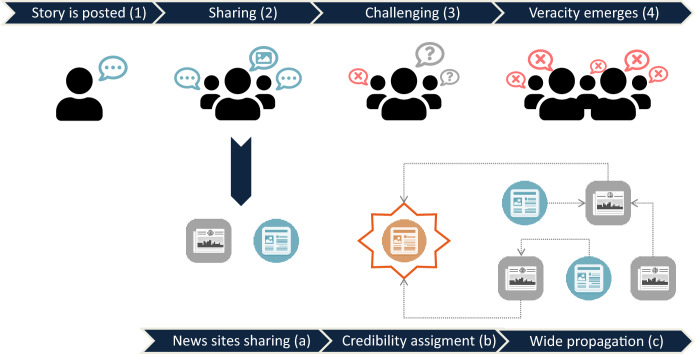
Disinformation and rumor propagation. The propagation generally starts when a social media user posts an alleged story (1). Then, the story might be shared with additional evidence (2). Other social media users begin to challenge the credibility of the story (3). After some time, a consensus about the veracity of the story emerges (4). During the sharing phase, the story may be caught by small outlets which spread this narrative (a), assigning a credibility stamp to it (b). Larger news sites are now encouraged to replicate the story until it is no longer possible to find the original source (c)

Usually, a story first debuts in social media posts (Fig. 1). Then, it is shared and some form of evidence (e.g., pictures, eye-witness reports) may be added as it spreads. In this sharing phase, various reformulations may also happen. At some point in this cycle, the credibility of the story starts to be challenged. Finally, in light of new and more consistent evidence, a consensus about the story’s veracity begins to emerge.

During the sharing phase of disinformation and rumor propagation, it may be caught by small or local news outlets, without being fact-checked due to lack of staff, financial restrictions, or other motives. Then, a vicious cycle begins [42]: some of the news sites repeating the story opt to employ headlines reinforcing its veracity, aiming for clicks and sharing, while others merely use expressions like “*reportedly*”. This gives the claim a credibility stamp, which encourages mid-sized and national news outlets to propagate the story, always pointing to previous spreaders and eventually adding some context. Once the claim is widespread, it becomes impossible to locate the original reference amid several interlinked news articles. Then, as the story is repeated by many sources, belief effects on readers are hardly countered.

This trajectory from an initial social media network post into a widely disseminated news story may occur within minutes or hours after a claim is published [42]. However, the mainstream media eventually release delayed follow-ups in relation to the crowdsourced stories posted in social media [40].

## Cognitive vulnerabilities

Post-corrections and denials may fail to completely eliminate the impressions caused by misleading messages, especially if individuals have preexisting attitudes and formulated opinion about a subject [43]. Even more serious, when news sources try to report false information cases, they may unintentionally give them even more exposure. Beliefs and rumors, even when discredited, “still tend to persist, but in a weakened state” [44]. Hence, rebuttal resistance directly affects journalistic and mainstream media daily work and put fact-checkers under skepticism [45]. Media manipulators also recognize these cognitive effects and take advantage of them. For manipulators, it is indifferent whether the media is reporting on a story in order to debunk or dismiss it, as long as they get the story covered [7]. It is essential that their story is widely commented, so that they are acknowledged as influential players. In summary, if manipulators succeed in framing a story from the beginning, their version is more likely to be assimilated by the audience.

Cognitive sciences have been trying to understand the psychological factors that make people more vulnerable to misinformation [46]. Different studies explore people’s difficulties in recognizing false narratives or even the challenges caused by belief perseverance phenomena that hinders misinformation correction [42, 46]. These phenomena usually do not occur in isolation but may complement each other or even obey a cause-and-effect relationship. Table 3 briefly describes ten cognitive phenomena strictly related to belief perseverance that can be exploited for spreading misinformation.

Under the influence of cognitive biases, social media accelerate the proliferation of information, potentially stimulating extremism and the spread of misinformation [32, 47]. If, on the one hand, social media users can control the dynamics of their connections, constituting segregated and polarized clusters, or “echo chambers” [48], on the other hand, social media content is usually delivered to users in a personalized fashion, i.e., information is automatically filtered and prioritized, based on data collected from individual users’ interactions, preferences, and interests. The intentional or algorithmic-determined exposure of users to personalized content results in a more frequent interaction between individuals with similar views and preconceptions, leading to the creation of “filter bubbles” [49]. These filter bubbles are therefore organically nourished by members of the community, who provide and disseminate information compatible with the group’s interest so that there is no longer a need to resort to external sources (e.g., Facebook groups or, 4chan community). Such interactions reinforce polarization by stimulating beliefs common to group members.

Ideological polarization is often perceived as a threat to democracy. As a consequence of polarization, the proactive debate is discouraged in order to maintain the group identity and homogeneity (*groupthink*) [50]. Combined to the effects of repeated exposure, group polarization pushes people to hold shared views with more confidence [51], disregarding potential alternatives for a problem resolution [52]. This creates a dispute of narratives, in which like-minded individuals are encouraged to have a negative view towards the opposing ideological groups or external perspectives [47, 53]. Remarkable events are pointed out as the effects of group polarization, as the 2016 US presidential election, and 2016 EU referendum in the UK [54]. However, these are not isolated cases, but portraits of a global crisis. McCoy et al. illustrate common patterns in different countries, such as the democratic erosion in Hungary, and a growing authoritarianism in Turkey and Venezuela [55].

**Table 3:** Cognitive vulnerabilities that can be exploited for spreading misinformation

Confirmation bias	The act of searching evidence to support existing bias or expectations [56]. Also referred to as selective exposure, this phenomenon makes us blind to information that contradicts our beliefs in order to minimizing cognitive dissonance [42].
Motivated reasoning	The tendency to “scrutinize ideas more carefully” if we do not agree with them [57]. In other words, our ability to reason is unconsciously affected by our preexisting values, identities, and attitudes [42, 58].
Biased assimilation	A process related to motivated reasoning in which people interpret new information in a biased way, in accordance with their own beliefs [42]. This phenomenon explains why individuals “readily accept confirming evidence while critically examining disconfirming evidence” [59].
Hostile media effect	An effect related to the bias perceived by individuals from their preexisting stance towards the news source. Because of this effect, people with opposing views, when accessing the same reports, tend to perceive these reports as biased against their own opinions [60, 61].
Repeated exposure	Repetition leads to familiarity and people use familiarity as a proxy for credibility. It increases the processing fluency (the ease of information recall), which is perceived as discrepant from a comparison standard and may affect truth judgments [62, 63].
Denial transparency	This phenomenon portrays the ineffectiveness of denying a proposition. It is attributed to the way people cumulatively process information, always appending new pieces to their “store of knowledge”, without deleting previous information [64].
Backfire effect	This effect highlights the increase of people’s acceptance of challenged beliefs when presented to contradictory evidence [65]. It may occur as a result of repeated exposure.
Group polarization	It is explained through the predictably behavior of group members adopting a more extreme stance after group deliberation [51]. Groups of like-minded people reverberate messages, such as in an echo chamber, with a social function to legitimize each other, reinforcing individuals’ opinion [66].
Causal inference making	The act of attributing unwarranted cause–effect relationships to contiguous events. After the occurrence of an event, people tend to mistake their inferences with real memories of the event, yielding auto-suggestion errors [67].
Emotion	Previous research indicates that the accuracy of personal beliefs and resulting attitudes can be shaped by a person’s emotional state and by the prevalent tone of media coverage [46, 68].

Strategies to penetrate these social bubbles and expose their members to diverse narratives are scarce. Recent mitigation theories have been proposed to prevent the emergence of echo chambers on social media. Sasahara et al. [48], for example, argument that a possibility would be to expose users to content distant from their preferences, e.g., by discouraging triadic closure when recommending new social ties, using algorithms to optimize the diversity of opinions, and preventing the complete dissolution of ties with dissonant users. However, these strategies seem vague and totally dependent on proprietary algorithms whose knowledge is not available for public scrutiny. More work is certainly needed to investigate how to break these already established segregated social clusters.

By knowing the cognitive phenomena, information consumers can explore strategies to deal with such vulnerabilities when avoiding misinformation. Many of the described cognitive phenomena can be neutralized through educational approaches that encourage media literacy and self-knowledge, seeking to make consumers aware of their own bias. Furthermore, the reinforcement of journalistic practices that uphold a fair coverage and a greater diversity of views on a given topic also help countering such vulnerabilities. On top of these, digital platforms and computational solutions can support the decision-making process of whether or not to consume a news piece. Table 4 describes the different natures (autonomous, mediated by third-party agents or completely controlled) of this decision-making process.

## Strategies for coping with misinformation

Misinformation permeates different social spheres, bringing concerns and losses of multiple natures. Since misinformation is a multidisciplinary problem, it also demands solutions that contemplate the diverse points of view of the most varied domains. This section presents an overview of the different perspectives for addressing misinformation, whether they are focused on journalistic, educational, governmental, digital platforms, or computational solutions.

### Journalistic perspective

Journalism is living an epistemic crisis strictly related to the technological advances in media manipulation, “which alter previously established notions of what a trustworthy source is” [69]. Journalism is now faced with a new paradigm, in which falsehoods are produced and spread along with accurate information, demanding journalists to reinforce and to renegotiate their roles as truth-oriented dissemination agents, to be perceived by the public as reliable sources of information [70].

**Table 4:** Characterization of decision-making processes

Autonomous	Solutions to assist autonomous decision-making process leave the news consumption decision completely up to the reader. These solutions aim to develop literacy so that the consumer is empowered to judge the quality of the available content.
Mediated	In mediated consumption, the available information is partially curated by third-party agents, such as journalists or algorithms. These solutions are designed to facilitate evaluation tasks. Although mediation solutions present inputs that can be useful in the assessment, the final verdict will always be issued by the consumer.
Controlled	In controlled decision-making process, solutions curate the available information without considering the consumer. This process includes pre-established news analysis or even the omission of news content deemed by third-party agents to be malicious, dangerous, or inappropriate.

The lack of journalistic rigor may allow misinformation to originate from or penetrate newsrooms and be then broadcast by the official channels of news organizations. Poor quality journalism arises from faulty research, sloppy verification or even by manipulation practiced by actors in defiance of the ethics of journalism [71]. The inadequacy of newsroom resources, editorial agenda, and the pressure for an ever wider and immediate coverage of events also contribute to poor quality journalism [72]. The demand for covering unfolding stories pushes journalists, imposing temporal restrictions for an in-depth investigation or even for revisiting previous works [73]. This lack of internal curation delegates the verification activity to external actors who are able to point the mistakes made by journalists. As a result, the general public begins to question the legitimacy of newsrooms and to attack the credibility of journalists [70, 71].

Dealing with massive amounts of misleading content requires journalists and news organizations to reinforce traditional good practices of journalism and to adopt new ones. Every source, and consequently, every piece of information, has a tendency and thereby a potential bias. Thus, a careful investigation should figure out the source’s origin, the source’s leaning and compare each narrative to alternative versions [69]. By scrutinizing information sources, journalists make information tendencies transparent when delivering information to the general public.

Fact-checking is becoming popular as an initiative of unbiased journalism, establishing standards and practices to assess the veracity of public claims [74]. As stated by the American Press Institute, “fact-checkers investigate verifiable facts, and their work is free of partisanship, advocacy, and rhetoric” [75]. Most fact-checking initiatives operate outside traditional news organizations, having institutional links with the academia and politics/civil society, but maintaining journalism as their core [74]. Experimental studies highlight the corrective potential of fact-checking, especially in polarized environments. Hameleers and van der Meer showed that partisans, when exposed to fact-checking, were able to reconsider their pre-existing misperceptions and moderate their attitudes [76]. Also noteworthy, the delivery format of corrective information (text, audio, and visual) plays a significant role in belief-correcting effectiveness, with video messages being more successful [77].

Although there is no consensus on how fact-checking affects beliefs [78], its relevance is already recognized by the audience. Wagner and Boczkowski conducted a series of interviews, with participants diverse in socio-demographic categories, to investigate the routines of news consumption [79]. The authors identified that, to counter perceptions of misinformation consumption, individuals often resort to traditional fact-based media and fact-checking services.

The reliance on fact-checking to combat misinformation, however, presents important limitations: (1) it fails to address the majority of information consumers that receive news exclusively through social networks and do not subscribe to traditional media sources; (2) fact-checkers have a limited capacity and cannot manually cover everything that is propagated through media channels [41]; and (3) misinformation is often more viral and generally spreads faster than corrective information, besides being a more interesting narrative [7, 42].

Other research studies advocate strengthening journalism through interdisciplinary strategies, relying on media and information literacy for empowering individuals to distinguish quality news from misinformation [71]. Examples of such partnerships include journalistic, governmental, and business organizations, such as the Facebook Journalism Project, which promotes collaborations with publishers around the world. One example is a collaboration with Reuters, which released an e-learning course on helping newsrooms around the world spot deepfakes and manipulated media [80].

### Educational perspective

Another strategy to cope with misinformation involves educating individuals to consume information produced by the mass media. The complexity of promoting conscious consumption of information lies in the fact that people are flooded with content all the time, not being able to properly select and to process the information consumed. Saturated with mass messages, consumers feel overwhelmed and try to protect themselves by narrowing down their focus and filtering out more messages [81]. Amid this process, people become vulnerable to media manipulation. It is no longer enough to just have better-trained journalists and professional gatekeepers, but to invest in the generation of educated news producers, distributors, and consumers, who know how to be their own editors and are able to identify fact-and-evidence-based news and information [82].

The attempt to educate the consumption of information and the way individuals interact with the social and mass media brought to light two concepts: information literacy and media literacy. The nature of information literacy emphasizes the ability to recognize when information is needed and the careful selection of information in specific domains and contexts, oriented by critical thinking, meta-cognitive, and procedural knowledge [83]. On the other hand, media literacy has been broadly defined as the ability to access and understand different aspects of the media, critically evaluate media contents, and create messages in a variety of contexts [84].

The traditional information and media literacies education takes place in classrooms, where a set of concepts are introduced and discussed, e.g., the role of journalism in creating news, the critical news consumption, and the importance of interaction between the press and the public [85]. These literacies also have a highly interdisciplinary character, highlighted by the use of methods and tools from sociology, psychology, political theory, gender and race studies, cultural studies, art, and aesthetics [83].

Previous research has demonstrated that media literacy education can be substantially beneficial. Jones-Jang et al. empirically investigated whether individuals with greater literacy were better at recognizing disinformation [86]. The authors found a significative association between the accurate identification of falsehoods and information literacy. Kahne and Bowyer studied how are assessments of truth online claims influenced by individuals’ exposure to media literacy learning opportunities [87]. For this, young participants were asked to rate the accuracy of evidence-based posts and posts containing misinformation. The authors verified that individuals with high levels of media literacy learning opportunities were “more likely to rate evidence-based posts as accurate than to rate posts containing misinformation as accurate”. In contrast, the same was not verified with individuals who were not exposed to media literacy education.

Some authors, however, are more reticent about relying exclusively on information and media literacy [46, 88]. Although literacy is recognized as a helpful mechanism to cultivate critical media consumption, it must be repositioned to contemplate a digital era of partisanship and distrust [89]. We can already spot new trends towards the use of computational tools and campaigns to foster literacy in the online environment (see “Digital platforms' perspective”, “Computational perspective”, respectively). In a proof of concept, Basol et al. demonstrated that the promotion of media literacy through educational games is effective in inoculating both specific instances of misinformation and strategies used in its production [90].

The educational strategy, although promising, is more focused on the long term, given that the construction of a capacity for critical thinking takes time to mature in society. On the other hand, the effects of misinformation inoculation through digital literacy may have an individual durability restricted to a few weeks, requiring constant and recurring training of people’s ability to distinguish false content [91]. Educators must also be aware of a potential trade-off between perceived media accuracy and media literacy, as mainstream stories could be viewed more skeptically by literate consumers [91].

Educational approaches still fail to consider structural problems in education due to the idiosyncrasies of each social group. They also presuppose the active participation of government agents working in education, which can be problematic in authoritarian governments that benefit from the population’s ignorance and have no interest in increasing the educational levels of the countries they rule. Therefore, people with less access to education will have a harder time building critical thinking. As educational approaches aim at long-term changes, the population remains vulnerable to misinformation until information and media literacies are fully built.

### Governmental perspective

Government actions to deal with misinformation are generally based on legal strategies, whether punitive or regulatory. Among these are social media moderation, application of steep fines, and even the imprisonment of company leaders or misinformation spreaders [92]. In April 2019, the Law Library of the US Congress examined existing legal approaches to handle disinformation through mass and social media and the legislative measures undertaken to counteract its proliferation [93]. This study identifies three approaches that can be used individually or combined:

- In the absence of specific disinformation legislation, some countries apply provisions of existing laws regulating the media, elections, and anti-defamation, even though these laws fail to reflect current technological advancements [93].
- Application of new and more focused legislation imposing sanctions on social media networks spreading disinformation (e.g., fines or orders to remove content identified as false).
- Provision of the means for authorities and digital platforms to secure a well-informed audience, either by identifying and blocking false news, offering fact-checking resources for the general public, or through the mass publication of reliable news [93].

While many countries have been taking legal action to counter misinformation, regulation is still a sensitive issue. In March 2018, the European Commission published a report to advise on policy initiatives to deal with disinformation spread online [94]. The report addresses general objectives, emphasizing non-regulatory responses, “as government or EU regulation of disinformation can be a blunt and risky instrument”. This is a concern similar to that expressed by Brazilian fact-checkers, who are reticent about the “The Brazilian Law on Freedom, Responsibility and Transparency on the Internet”, approved by the congress in June 2020 to combat disinformation. According to Poynter Institute [95], Brazilian fact-checkers are concerned with the potential creation of a massive surveillance network, which would focus on journalists and activists, making the bill vulnerable to abuse and constituting a threat to data privacy.

In fact, regulatory acts sustain heavy criticism for being perceived as censorship and an attack on the freedom of speech. In Singapore, for example, the controversial Protection from Online Falsehoods and Manipulation Act (POFMA), allows government ministers to arbitrate and put warnings next to social media posts considered to be false. The POFMA has allegedly been used to silence opposition in the face of upcoming elections [96].

In China, a country known by strict media regulation [97], misinformation is a recurring concern, causing the government to take specific actions to curb the dissemination of online false news over the past few years. In 2016, the Cybersecurity Law criminalized manufacturing or spreading of false news that seriously undermine public order through an information network or other media, with a prison sentence of up to 7 years [98]. A year later, the Chinese government ordered network operators to monitor the information disseminated by their users. If any kind of contravention of administrative regulations is identified, the network operator is responsible for filtering and regulating, keeping relevant records, and reporting them to government authorities. Also, social media platforms must obtain a license to operate and authenticate their users using real names and additional identity information.

Enforcing strict content moderation can have disastrous consequences. The Chinese case shows that the lack of counteracting narratives affects consumer perceptions, leaving room for misinformation around dominant topics and polarization in the digital ecosystem [99], in turn leading to increases in pro-moderation or pro-censorship views [100]. On the other hand, there is a general concern that censorship may delay information on emerging critical topics, and therefore potential countermeasures, such as in the coronavirus outbreak in Wuhan [101, 102]. Imposing restrictions, however, is not synonymous with effectiveness. In response to regulations, Chinese citizens have acquired a proficiency in bypassing censorship, developing greater flexibility in learning new tools, and finding new resources to expand their knowledge [103].

In summary, regulation of the information landscape is quite fragile and prone to backfire, raising four central issues: (1) legal mechanisms may be used as an instrument of abuse and oppression; (2) even if regulation is not effectively designed to censor, it can be perceived as censorship, generating general dissatisfaction among citizens and a loss of trust in government entities; (3) individuals will eventually find ways to bypass regulations; and, ultimately, (4) the use of strict regulation can inhibit dissent voices and foster misinformation.

### Digital platforms’ perspective

The advent of digital platforms has lowered communication barriers, providing the public discourse a wider range. Platforms play an important role, either as channels or catalysts for information manipulation. Eventually, the companies operating these platforms benefit financially from this game by boosting misinformation and facilitating bad actors to reach a large audience. As these actions have become more frequent and increasingly explicit, digital platforms were criticized and put under pressure to act. For instance, in September 2018, representatives of online platforms, leading social networks, web search engines and other stakeholders signed the Code of Practice on Disinformation published by the European Commission [104]. Among the signatories are organizations like Facebook, Google, Microsoft, and Twitter. The Code of Practice is an agreement including a range of commitments, from “further transparency to the closure of fake accounts and demonetization of purveyors of disinformation”.

Lately, we have seen a shift in the stance of digital platforms, with greater collaboration with academia, government, and industry to avoid media manipulation. Table 5 lists some policies adopted by leading companies to curb misinformation. The most common actions include content moderation, partnering with fact-checking networks, promoting quality news while reducing the visibility of websites that share false news and making scams unprofitable. The Election Integrity Partnership (EIP), focused on understanding misinformation in the social media landscape during the 2020 US presidential election, identified three major moderation practices adopted by digital platforms [105]: (1) removing content and suspending accounts due to inauthentic identities and/or behavior, or repeated violation of the community guidelines; (2) reducing the distribution of policy-violating content, e.g., downranking content or preventing sharing capabilities; and (3) informing users about the veracity of the content through fact-checking labels.

The EIP also reported a number of updates to election-specific policies announced by platforms, such as Facebook, Twitter, YouTube, TikTok, during 2020 [105]. In particular, these updates focused far more on explicit topical content restrictions (e.g., claims of premature victory and promotion of violence at the polls), than on user behavior (e.g., foreign interference and coordinated inauthentic behavior). The partnership then found that platforms’ interventions shape users’ tactics, and users tactics impact platforms’ policies. From this observation, we can notice that platforms are likely to adjust their policies to the new practices of violation. On the other hand, offenders also adapt to the new restrictions, making it difficult to contain the spread of misinformation.

**Table 5:** Policies adopted by leading companies to curb misinformation

Facebook	Investments in partnerships with journalists, academics, and independent fact-checkers, to reduce the spreading of misinformation [106]. Actions focused on: (1) removing accounts and content that violate Community Standards^a^ or advertising policies; (2) reducing the distribution of false news and inauthentic content and users; and (3) giving users more context on the posts they see.
Google	Launch of the Google News Initiative^b^ to fight misinformation and support journalism, based on three pillars: (1) increasing the integrity of information displayed, especially during breaking news or crisis situations; (2) collaborating with the industry to surface accurate information; and iii) helping individuals to distinguish quality content online through media literacy.
Microsoft	Creation of advertising policies to prohibit “ads for election related content, political parties and candidates, and ballot measures globally”; application of these policies to Microsoft services, such as Bing and LinkedIn; partnership with NewsGuard [107] to provide a browser plug-in to warn users of untrustworthy news sites.
Twitter	Political advertising ban^c^; interactions with the public to jointly build policies against media manipulation [108]; labeling and adding warning messages to misleading posts to provide additional explanations or clarifications [109].

^a^https://www.facebook.com/communitystandards/

^b^https://newsinitiative.withgoogle.com/

^c^According to Twitter CEO Jack Dorsey, political advertising forces “highly optimized and targeted political messages on people”, which brings significant risks as “it can be used to influence votes to affect the lives of millions”. See: https://twitter.com/jack/status/1189634360472829952

A very recent new trend is large companies asking digital platforms to be less permissive. In June 2020, the power and hegemony of social networks began to be challenged. Because of Facebook’s inability to curb misinformation and hate speech, a conglomerate of multinationals and civil rights groups organized the biggest corporate boycott in the company’s history [110]. The “Stop Hate For Profit” campaign calls on Facebook to have stricter measures against racism, disinformation and hate on its platform. The movement encourages businesses pausing paid advertising to press Facebook to make changes, such as providing more support to people who are targets of racism and hate, stopping generating ad revenue from misinformation and harmful content and eliminating large groups filled with hate conspiracies [111]. However, this movement faced some immediate resistance as Facebook refused to change its policies [112].

It should also be reinforced that digital platforms are entities with commercial interests, dependent on proprietary software and serving the interests of customers and other interested parties who inject revenue for the promotion of brands, products, or content and for the manipulation of public opinion. Therefore, the limitations related to this perspective are very similar to the problems generated by the governmental perspective. There is no guarantee that the discourse of the representatives of these platforms will be aligned with their practices. Since the algorithms implemented by these platforms are not publicly available, it is not possible to put them under independent scrutiny to evaluate their accuracy and to identify possible flaws [41]. Finally, we would always question their methods and impartiality.

### Computational perspective

Technological strategies for handling misinformation provide supporting tools for promoting media literacy and assisting the manual or automatic fact verification process. This section systematizes the most common roles played by computational solutions. However, some of the strategies employed by the described tools could be included in a more general misinformation classification framework (subsection “Automatic misinformation classification”).

#### Supporting solutions

Computational solutions are employed either as standalone tools or to assist and complement other approaches, such as educating consumers, supporting journalism, and improving digital platforms. Misinformation tools make use of machine learning models to infer the veracity of a news story or for automatic fact-checking. Misinformation indicators have also been employed by computational solutions as an assisting mechanism to help consumers to spot misleading content in online news sites and social media. Such tools may take as input assessments from crowdsourcing initiatives, journalists, and/or algorithms. Misinformation indicators are designed to provide consumers with insights on the quality of the news, so that consumers can decide by themselves whether to trust on it [113]. As an example, NewsScan is a web browser plugin that automatically computes and assigns a set of misinformation indicators to news articles [114]. The indicators are generated using different computational resources based on influence ranks, social media, and machine learning methods.

Computer-based solutions are aimed at wide audiences, from journalists and fact-checkers to even the general public. Consequently, their role may vary according to the target audience (see Table 6):

Education: Computational solutions that support the autonomous decision-making process rely on the target public’s capacity of evaluating the information quality. Examples of these solutions include tools to promote information and media literacy. For example, Google Interland^1^ and Bad News^2^ [115] are web-based games focused on teaching kids the fundamentals of critical thinking and digital citizenship, thus promoting media literacy and making the player aware of techniques used to spread misinformation.
Collaboration: Computational solutions can also foster collaborative approaches to deal with misinformation. These solutions are also heavily dependent on the autonomous ability of users to identify quality information. The target audience for these approaches can be both journalists/fact-checkers and end consumers of information. Examples of such solutions include tools for the communication of collaborative groups (e.g., Newstrition^3^) and verification platforms (e.g., Checkdesk^4^ and Truly Media^5^). Newstrition is a collaborative tool that provides misinformation indicators attached to news articles. Users can manually insert data about news publishers, article sources, fact-check analysis, media bias ratings, and other contributions. Checkdesk and Truly Media are verification platforms that allow journalists to monitor social networks and work either within their newsroom, or in collaboration with information consumers.
Assistance: Computational tools can also be used to evaluate news quality. Computer-assisted fact-checking tools aim at supporting journalists and editors with the curation and investigation of unverified content. Such material can come from different sources, including political speeches, live interviews, or user generated content. Automatic tools may rapidly detect factual claims in news text, track mentions of claims already identified as false, and check numeric claims against authoritative databases [41]. They can also analyze the propagation of false claims in order to spot the agents that are spreading false narratives and create fact-checks of unseen claims based on related trusted data. Full Fact’s Live platform^6^ and Chequeabot^7^ are examples of fact-checking tools designed to be used in newsrooms and by fact-checkers.
Communication: Once a news item or a news source has already been verified by trained experts, there are computational tools capable of communicating the evaluation outputs for the general public. This is the category where fact-checking websites and web services are found. Also included in this category are browser plugins with analysis conducted by specialists. NewsGuard^8^ is one such plugin that uses the ratings and reviews of trained journalists and experienced editors to evaluate news and information websites. Depending how a website is evaluated, it is assigned a color rating based on nine journalistic criteria grouped into two categories (credibility and transparency). The judgments provided by NewsGuard rely exclusively on human analysis rather than employing algorithms to identify unreliable news.
Decision: Automatic solutions depend solely on the judgment of algorithms to evaluate a news item. These can be trained on previously evaluated datasets, but there is no human interference in the decision process. Fully automatic computational tools support both fact-checking (e.g., ClaimBuster^9^) and the assessment of the news sources’ credibility (e.g., News Coach^10^). ClaimBuster uses Natural Language Processing (NLP) techniques to identify factual and false information [116]. News Coach uses algorithms to automatically detect websites, blogs and video channels and classify these sources into reliability categories.

**Table 6:** The role of computational solutions assigned to the corresponding decision-making process (DMP) and target audiences, followed by examples of existing tools for this purpose

Role	DMP	Target audience	Examples
Education	Autonomous	Consumers (general public)	Google Interland; Bad News
Collaboration	Autonomous	Journalists and fact-checkers; consumers (general public)	Newstrition; Checkdesk; Truly Media
Assistance	Mediated	Journalists and fact-checkers; consumers (general public)	Full Fact’s Live platform; Chequeabot; NewsScan
Communication	Controlled	Consumers (general public)	NewsGuard
Decision	Controlled	Consumers (general public)	News Coach; ClaimBuster

From the analysis of the services listed in Table 6, we infer that solutions for coping with misinformation are still in early stages of development. Little information about their use or scalability is available either in scientific literature, web application stores, or even in communities/profiles on social networks. Some of these solutions were designed as (or have become) paid services (e.g., Truly Media and NewsGuard), making it difficult to infer the number of users. As for the other tools, there are no references to large user communities around these services, with rare exceptions exceeding the 100,000-user mark (e.g., Google Interland, Chequeabot). An interesting exception is the game Bad News. Despite no indication of widespread adoption by the general public, the authors conducted an extensive assessment of the game’s effectiveness in inoculating falsehood spreading strategies, showing that the game is capable of creating a “cognitive immunity” to misinformation [90, 117].

#### Automatic misinformation classification

**Fig. 2:**
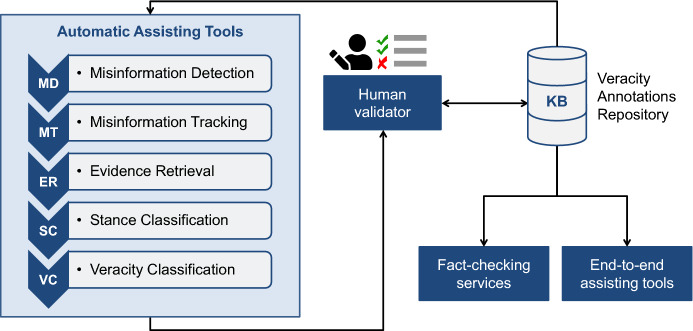
Architecture of misinformation classification system. Automatic tasks: misinformation detection, misinformation tracking, evidence retrieval, stance classification, and veracity classification. The output of the automatic pipeline is manually evaluated by a human validator. The human verdict is stored into a repository which delivers reliable material to fact-checking services and end-to-end assisting tools

The social concern about misinformation spread promoted the emergence of several initiatives to automatically identify and elucidate incorrect or unsubstantiated claims. Most of these initiatives rely on machine learning or NLP techniques for identifying and preventing the spread of misinformation. A misinformation classification system can be structured as pipeline consisting of five tasks (Fig. 2):

(i) *misinformation detection*, i.e., determining the pieces of information with an unverified narrative;
(ii) *misinformation tracking*, i.e., mapping the narrative repercussion and propagation by gathering related content published by different sources (or reactions from the audience);
(iii) *evidence retrieval*, i.e., identifying suitable textual evidence from reliable sources;
(iv) *stance classification*, i.e., determining the attitude expressed in the related material with respect to the narrative;
(v) *veracity classification*, i.e., estimating the veracity labels and, optionally the confidence score, of a narrative.

There is substantive research on misinformation detection in its early stages. This is a specific sub-task within the scope of *misinformation detection*. *Early Detection* is mainly conducted on the reactions elicited by a story on social media, by processing incoming posts as a data stream and monitoring the posts in real time [118]. *Early detection* systems incrementally utilize the available information observing a trade-off between confidence in detection accuracy and timeliness of the detection and mitigation effort [119].

Similar architectures were previously suggested. For example, Zubiaga et al. [15] organized a systematic pipeline to tackle rumor classification. However, this system may have missed a crucial step to assess the veracity of a news piece. Zubiaga et al. focused on applying this architecture to social media, but we argue that most of the sub-tasks could also be performed on different information sources, such as news outlets. Nowadays, news outlets and social media are closely interconnected. Publications made on news websites immediately reverberate on social media. Similarly, issues in the spotlight on social media may dictate newspapers' agenda. These dynamics are reflected in recent misinformation collections, such as FacebookHoax [4], BuzzFace [120], and FakeNewsNet [121], which combine stories published by news outlets and the respective developments in social media.

As pointed out by previous studies, automatic systems for fact-checking and information veracity classification have not reached a sufficiently high maturity level to operate without human supervision. Failures in the automatic classification can be very costly, either leading to the spread of misinformation (false negatives) or to censorship, by the invalidation of true stories (false positives) [41]. Therefore, we believe that an information classification system should be designed to provide tools to assist the decision-making process conducted by a reader.

Accordingly, Fig. 2 depicts the architecture of a misinformation classification system, which contains an automated part including all the processes defined in the misinformation classification pipeline. The final decision rests on a human validator, which would be supported by the outputs of the algorithms, in addition to a reliable repository, such as a knowledge-based (KB), of previously annotated resources. Once the human validator creates a new verdict, this information is also added into the KB. The KB can then store reliable content that serves as input for the automated processes performed by algorithms, fact-checking services, and end-to-end assisting tools, in addition to serving as a basis for future annotations made by humans.

The major limitation of current computational approaches is that they hardly address the needs of the news consumer. Many of the news veracity assessments do not accompany supporting evidence. And even the misinformation indicators may not be transparent to consumers. These two factors make it hard for consumers to engage with computational solutions. Other two structural limitations of the existing computational solutions are scalability and outdated models. The scalability issues occur mainly because a large part of the existing tools depends on human judgment to manually assign labels that are used to train models based on machine learning and deep learning techniques. Such models are heavily dependent on a massive amount of data to produce good-quality automatic classifications. In addition, models trained on old datasets would be unable to learn new instances of misinformation strategies and would therefore need to be periodically retrained with fresh datasets [41].

## Discussion

Considering the strategies for countering misinformation presented in the previous section, now our discussion focuses on how such strategies can be combined using multidisciplinary approaches, and on the gaps in knowledge within each perspective and between the various perspectives.

### How do computational solutions interplay with the other perspectives?

In this section, we highlight approaches that bring together computational solutions and offer strategies to combat disinformation. We cover in particular the use of machine learning-based approaches for the automatic classification of misinformation. However, this list is not exhaustive, offering only examples of how computational solutions could be developed or have been employed in the past. We organize the computational approaches suggested for each perspective according to the framework discussed in “Computational perspective”. It is also worth noting that two or more tasks in the presented misinformation framework can often be used together.

Journalistic and fact-checking activities can be enriched with the use of automatic misinformation classification mechanisms. For example, misinformation detection systems can help news agencies to early find (early detection) narratives that have been gaining wide notoriety among the general public. Once these stories are identified, journalists can work on their manual validation, investigating the sources, and checking the reported facts. Besides, automatic systems can also be employed to identify evidence capable of supporting or refuting disputed narratives. This would facilitate the verification work, speeding up this process and reducing the workload of those involved in fact-checking activities. Checkers can, therefore, resort to news repositories or other analysis already carried out by different agencies to substantiate their verification.

Digital platforms also benefit from collaborations with fact-checking agencies, such as Facebook’s Third-Party Fact-Checking Program, which allows verified signatories to the International Fact-Checking Network to flag posts, videos, and photos as false. In addition, digital platforms hold plenty of textual and visual data to train automatic models to identify misinformation (misinformation tracking) and stop chains of false news dissemination. Digital platforms can, therefore, employ tools to identify users (be they real accounts, cyborgs, or bots) creating or working on the spread of disinformation. As an example of these tools, the Observatory on Social Media (OSoMe) and the Network Science Institute (IUNI) developed Botometer^11^ [122], which analyzes Twitter accounts' activity and assigns a score indicating bot-like activity. Digital platforms might also resort to automatic approaches to offer users a greater diversity of perspectives and points of view. For example, given a thread on social media, stance detection approaches can be used to find comments that present a different perspective on the narrative that started the thread. Once such comments are identified, digital platforms should act to penalize bad content and/or highlight reliable comments, as has been done by Facebook [123].

The diversity of content provided by digital platforms and social media to information consumers also contributes to promoting greater media and information literacy. By giving voice to different narratives, social media challenge individuals’ cognitive biases, encouraging the disruption of filter bubbles. Computational solutions can also strengthen literacy by facilitating the exposure of individuals to contrasting perspectives, identified through stance detection and evidence retrieval models. Accordingly, such models help identifying relevant evidence from large collections of documents and reasoning about statements whose veracity is often not clear. For example, FEVER (Fact Extraction and VERification) shared task encourages the development of automatic systems capable of identifying evidence and inferring the veracity of human-generated textual claims from a wide structured repository of information, such as Wikipedia [124]. In addition to automatic classification tools, other computational solutions are also useful to foster media literacy. The aforementioned Bad News game consistently showed improvements in people’s ability to spot misinformation techniques [90].

Given the scalability restrictions of fact-checking conducted by human checkers, complementary classification models favor the awareness of lay news consumers. The tools currently available mostly employ two different strategies: the automatic classification of veracity or the presentation of credibility indicators. The first strategy includes issuing a verdict on the veracity of claims (e.g., ClaimBuster). In its simplest version, this approach uses fact-checking databases, accessed on demand by news consumers. More elaborate approaches employ classification strategies through models based on deep learning. The second strategy is to present a set of misinformation indicators (e.g., Newstrition, NewsScan). Similar to the nutritional information presented on the packaging of food products, the use of indicators representing evidence of unreliable information is encouraged [113]. Based on such indicators, readers would be led to refine their perception of suspicious features in news, such as the use of a strong emotional charge when reporting an event [125].

Government campaigns focused on the development of media literacy also contribute to greater resilience of individuals against misinformation. As an example, the European Commission launched programs and initiatives, such as #SaferInternet4EU, aimed at youngest users, and “Media Literacy for All”, focusing on strategic communication against disinformation and the development of awareness-raising [126]. Government campaigns may also include partnerships with news organizations or digital platforms, as in the initiative jointly promoted by India’s National Association of Software and Services Companies and WhatsApp. The interventions made by this initiative comprise education campaigns in different languages and media formats, and the training of approximately 100,000 volunteers, through in-person events and/or posts on social media to spot misinformation [91].

Regulatory actions, although controversial, must be used. Government entities cannot rely entirely on internal audit conducted by third parties. The lack of specific laws to deal with the spread of misinformation on social media through false profiles makes it difficult to frame the practice in the criminal sphere. However, regulatory actions must observe the same misdemeanors (e.g., crimes of misuse of image, false identity and even injury, slander, or defamation) that occur in the non-virtual world to prevent the spread of disinformation or the use of false accounts, fully or partially managed by robots, trying to replicate human behavior, and inspiring credibility or sensitizing other social media users. The transposition of laws from the real world to the virtual scenario has already been applied by some countries. An example of this move occurred in Brazil, with the condemnation of a far-right online newspaper for using false profiles to attack politicians, judges, and ministers [127]. The alleged collaborators who subscribe to the reports would, in fact, be fake profiles using modified photos of public figures. The complaint that gave rise to the conviction was conducted by the fact-checking agency Aos Fatos [128], showing, once again, how the joint action of several entities contributes to curb misinformation.

Rather than being restricted to regulation, government solutions also bring together journalistic agents to promote literacy through computational solutions. Among such initiatives is the project Contrafake,^12^ led by the Portuguese news agency LUSA, with the main goal of creating resources to protect and support communication professionals, citizens, and institutions against disinformation disseminated through digital information sources. Contrafake project comprises the development of machine learning-based tools for (1) creating a set of misinformation indicators; (2) uncovering information manipulation actions and cyber-attacks; and (3) early identifying “viral” processes. Moreover, together with the Portuguese National Cybersecurity Center, LUSA also launched an online course to help people understanding misinformation, fostering “a critical sense in the consumption of information” on the Internet and, allowing readers distinguishing reliable news from opinions.

### Knowledge gaps within each perspective and between perspectives

Indiscriminate use of the aforementioned solutions is not, however, a panacea for coping with misinformation; hence any solution should consider the specificities of both the audience and the communication channel. Worse than being ineffective, the misuse of these approaches can even reinforce misperceptions by activating a range of cognitive phenomena. Table 7 summarizes the advantages and limitations of each defensive perspective referred to throughout “Strategies for coping with misinformation”.

**Table 7:** Approaches and limitations of different perspectives to handle misinformation

Approach	Plus (+)	Minus (−)
Journalism	Fact-checking; increasing the literacy of journalists to avoid giving voice to false narratives.	Corrections fail to reach a significant segment of the audience; denial effects caused by cognitive biases may reinforce belief in false stories; manual corrections are limited in scale.
Education	Promoting information and media literacy in each individual.	Focus on the long term; fails to consider structural problems in education; may be disregarded by authoritarian governments; is expensive; requires retraining.
Government solutions	Definition of a clear boundary between information and disinformation; punitive or regulatory measures can contain the production and dissemination of disinformation.	Content in the boundary is hard to regulate; may be used to restrict freedom of expression; may be perceived as censorship; lacks extraterritorial application; can inhibit dissent voices; individuals will find ways to bypass regulations.
Digital platforms	Enforcing moderation of content and transparency of advertising; promotion of quality news; partnerships with fact-checking agencies.	Vulnerable to commercial interests of customers and partners; dependent on proprietary software; opaque moderation process may be perceived as biased.
Computer Science	Usage of computational resources to support the automatic fact-checking and misinformation detection; development of misinformation indicators for promoting media literacy.	Generally, fails to address the consumers’ needs; may lack transparency; data annotation for training models is not scalable; models are quickly outdated.

Joint actions must be well coordinated and have clear objectives, otherwise they can lead to disagreements between players. For example, Facebook’s fact-checking program has sparked skepticism from partner organizations. The criticisms range from the terminology and categories used by the social network to rank disinformation to concerns regarding transparency on the impact of the fact-checking that independent checkers do [129]. Full Fact, an independent partner in Facebook’s project, published a report asking for answers to questions such as “Does the notification stop many people from sharing?” and “What percentage of people who view a post we have rated click on our fact check beneath it?” [130]. The same questions prompted Snopes, a US-based fact-checking agency, to end the partnership with Facebook [131].

The surveillance and control of users’ behavior on digital platforms still face difficulties when managing fake profiles and false narratives. First, platforms are progressively learning how to clearly and transparently communicate policies and interventions to the public. Then, although policy violations may result in an account removal, such measure is not effective since the offending user can circumvent this penalty by creating a substitute account [128]. To evade punitive moderation, political activists and influential profiles may either operate via sharing misleading messages published by fictitious accounts (i.e., acting merely as repeaters), or resort to cross-platforms sharing practices (i.e., propagating content from other websites and social media platforms) to limit the efficacy of any single platform’s response [105]. Due to the lack of uniformity in policies across platforms, it becomes complex to identify and punish influential spreaders and, even if the false narrative is removed from a platform, it continues to spread through other social media.

Another growing concern regarding digital platforms refers to the response to content moderation carried by mainstream platforms. Rather than containing extremist discourse, moderation can boost the migration of users to unregulated spaces or alternative forums, such as Parler, Gab, and Discord [105], Tor-protected websites, and closed messaging and communication services, such as WhatsApp, Signal, Telegram Messenger, and ProtonMail [9]. These platforms hamper access to data by reducing the effectiveness of regulatory actions and making the inoculation of falsehoods through complementary strategies, such as fact-checking or computer-based solutions, impracticable. In fact, messaging applications that resort to end-to-end encryption are becoming prevalent as disinformation channels [132].

The imposition of punitive measures on disinformation disseminators is also a point of conflict between digital platforms and governments. The 2021 permanent suspension of the accounts of former US President Donald Trump on Twitter and Facebook shed light on this discussion. While the actions of these platforms were motivated by the risk of further incitement of violence promoted by Trump, the German and the French governments pointed out this decision as problematic, claiming that restrictions on freedom of speech should be decided by the state, and not according to a private company [133]. On the other hand, in 2020, the French government itself passed a law regulating the removal of manifestly illicit content from social networks. Companies like Facebook, Twitter, YouTube, Instagram, and Snapchat must delete offending content within 24 h, or face a fine over their global revenue. Again, societal groups in favor of freedom of expression have spoken out against this deliberation [134].

When manipulations are effectively identified, it is difficult to punish the authors. One of the challenges in combating this type of crime is precisely to find those responsible for it. Some strategies to maintain anonymity include the abuse of protection services offered by companies that provide web hosting, which consist of masking the use of IPs or the use of extraterritorial web domains, preventing servers from being obliged to provide the identification data of the person responsible by a website when local legislation is triggered [128].

Also sensitive is the use of automatic tools as a means of detecting false and/or misleading content. There are no ethical guidelines on what data can be used to train models, what claims should be checked, or how these tools can influence public opinion. In the meantime, misinformation classification models need a massive amount of real data to be trained. It is a common practice of machine learning researchers to collect data from social networks, not necessarily with the consent of the private platforms or the individuals who produced that data. However, the ethical standards and practices that cover privacy protection are subject to intense debate, with differing interpretations, hindering the collaboration between universities, private companies, and organizational entities [135].

Additional research on the communication of decisions by automatic models is still necessary, mainly focusing on how end users, at different levels of educational background and digital literacy, interpret the results of such models. Automatic models will always be subject to failure, and the consequences of a wrong verdict can strengthen erroneous narratives, consolidate polarization and conspiracy theories, or unfairly discredit sources. The blind use of these tools by government entities or social media can also result in penalties for innocent individuals. Furthermore, recurring errors in the judgment issued by detection models would lead users to discredit and abandon the use of automatic tools. Ordinary citizens may not yet be prepared to deal with algorithmically driven information environments [135], stressing the importance of educational initiatives.

## Future outlook

Throughout this review, we have outlined the different nuances in which misinformation manifests. We were also able to understand how difficult it is to catch misinformation in its early stages of development, as well as to remedy the effects caused by exposure to it. The main goals were to introduce the different approaches to deal with misinformation and their limitations.

This review highlighted the limitations of different perspectives and, in particular, the difficulties in overcoming cognitive phenomena that reinforce belief perseverance. New strategies for delivering a solution that leverages the use of computational resources and focus on the education of news consumers and on their needs may represent alternatives (Table 8 presents suggestions of measures to deal with the cognitive phenomena described in “Cognitive vulnerabilities” as a way to combat misinformation). Furthermore, the scientific knowledge that would allow the evaluation of new methods to counter misinformation is still scarce. Only recently, studies capable of measuring the efficiency of such approaches have appeared in the literature (e.g., [91]). Given the limitations of all the surveyed approaches, only a cocktail of different strategies applying multiple technologies may lead to a more effective solution when dealing with misinformation. Consequently, technological approaches benefit from strategies that combine solutions based on individual skills with “solutions that address individuals’ (lack of) motivation to seek out, consume, and interpret information in ways that privilege accuracy over other possible goals, such as the protection of their preexisting beliefs” [46].

**Table 8:** Examples of measures for dealing with the cognitive vulnerabilities as a way to combat misinformation

Vulnerability	Defense	Example
Confirmation bias & Repeated exposure	Digital platforms & Computer science	Social media and web engines could adapt their algorithms to expose users to a greater diversity of narratives, reducing the existence of filter bubbles. We suggest the use of stance detection methods to identify divergent texts, and the redesign of digital platforms’ interface, prioritizing the balance of opinions. A new presentation way could, for example, show conflicting viewpoints side by side when exhibiting disputed stories.
Motivated reasoning & Biased assimilation	Education & Journalism	Literacy approaches could be used to make readers aware of their cognitive biases, encouraging a critical thinking and the reader engagement with a broad range of content. Educational strategies should also focus on teaching readers how to differentiate factual texts from opinionated material and on raising awareness of bad journalistic practices, such as the use of clickbait, personal attacks, or fallacies.
Hostile media effect	Journalism & Education	News outlets could represent significant views fairly, proportionately, and, as far as possible, without editorial bias [19]. The ethical commitment must guide journalistic conduct, and, more than ever, these professionals must act as gatekeepers, investigating and denouncing individuals and institutions that manufacture untruths.
Denial transparency & Backfire effect	Computer science, Education & Journalism	Computer scientists should keep in mind that most individuals do not understand how machine learning models work. Thus, rather than presenting opaque verdicts on news veracity, computational solutions should offer explainable and interpretable misinformation indicators [136]. The best way to present the outcomes of the decision-making process should be discussed with educators, journalists, psychologists, social scientists, and UX experts.
Group polarization	Digital platforms, Computer science & Education	Social networks can use machine learning algorithms to identify filter bubbles and monitor the visual or textual material shared in these groups. Once the intensification of polarization is detected, platforms should apply educational campaigns aiming to promote dialogue, but also considering the specificities of each group.
Emotion	Digital platforms & Governmental solutions	Social media could regulate and curb hate speech by limiting the influence of polarizing content, and restricting the exposure and reach of hateful material. Digital platforms can be even more proactive in combating this type of practice, alerting legal authorities about crimes of slander and defamation, and providing legal evidence when necessary. For this, we recommend the creation and/or expansion of compliance programs in private companies, observing local and extraterritorial legislation.

Rather than fostering misinformation solutions with regulatory or moralistic characteristics or approaches sounding like censorship or attacks to freedom of expression, we advocate the adoption of solutions supporting the strengthening of journalistic approaches for producing and delivering news content, while regaining public trust in news organizations. Thus, when accessing news items, readers would be sufficiently supported by informative references and able to conclude for themselves about the benefits and drawbacks of consuming bad content.

## Data Availability

Not applicable.

## Footnotes

1 https://beinternetawesome.withgoogle.com/.

2 https://getbadnews.com.

3 https://www.freedomforuminstitute.org/first-amendment-center/newstrition/.

4 https://meedan.com/en/check/.

5 https://www.truly.media/.

6 https://fullfact.org/automated.

7 https://chequeado.com/automatizacion/.

8 https://www.newsguardtech.com/.

9 https://idir.uta.edu/claimbuster/.

10 https://news-coach.com/.

11 https://botometer.osome.iu.edu/.

12 https://combatefakenews.lusa.pt/.
